# DNA Methylation and Recurrent Pregnancy Loss: A Mysterious Compass?

**DOI:** 10.3389/fimmu.2021.738962

**Published:** 2021-10-21

**Authors:** Qi Zhou, Yunhe Xiong, Bing Qu, Anyu Bao, Yan Zhang

**Affiliations:** ^1^ Reproductive Medical Center, Renmin Hospital of Wuhan University, Wuhan, China; ^2^ Urology Department, Renmin Hospital of Wuhan University, Wuhan, China; ^3^ Department of Clinical Laboratory, Renmin Hospital of Wuhan University, Wuhan, China

**Keywords:** DNA methylation, recurrent pregnancy loss (RPL), maternal-fetal immune microenvironment, epigenetic, immune

## Abstract

Recurrent pregnancy loss (RPL) is a common and severe pathological pregnancy, whose pathogenesis is not fully understood. With the development of epigenetics, the study of DNA methylation, provides a new perspective on the pathogenesis and therapy of RPL. The abnormal DNA methylation of imprinted genes, placenta-specific genes, immune-related genes and sperm DNA may, directly or indirectly, affect embryo implantation, growth and development, leading to the occurrence of RPL. In addition, the unique immune tolerogenic microenvironment formed at the maternal-fetal interface has an irreplaceable effect on the maintenance of pregnancy. In view of these, changes in the cellular components of the maternal-fetal immune microenvironment and the regulation of DNA methylation have attracted a lot of research interest. This review summarizes the research progress of DNA methylation involved in the occurrence of RPL and the regulation of the maternal-fetal immune microenvironment. The review provides insights into the personalized diagnosis and treatment of RPL.

## Introduction

**Figure 1:**
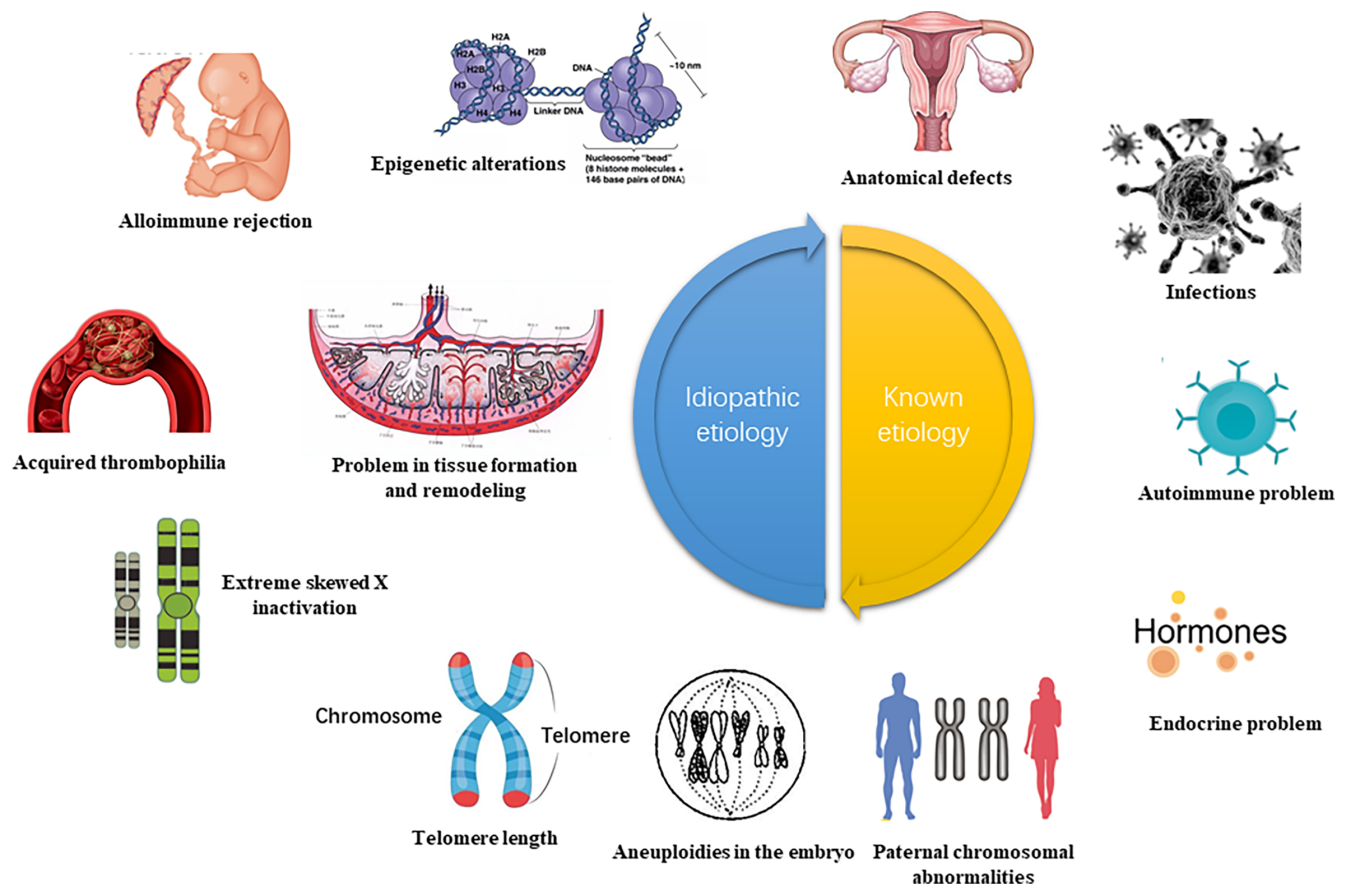
High risk of RPL (recurrent spontaneous abortion) (5). The etiology of RPL can be diagnosed by interrogation, clarifying the gestational age and characteristics of the miscarriage. Early RPL may be related to abnormal chromosome structure and function, immune dysfunction, endocrine disorder, abnormal reproductive system, prethrombotic state (PTS), poor environment and living habits. The function of the uterine cervical, the development of amniotic fluid, and the umbilical cord may also be linked to late RPL. This review focuses on the abnormal immune function of RPL and analyze the important role of DNA methylation.

Recurrent pregnancy loss (RPL) refers to three or more consecutive spontaneous abortion with the same spouse before 28 weeks of gestation. This complication affects about 5% of women of childbearing age globally (1). The latest epidemiological data released by the American Reproductive Medicine showed a spontaneous abortion prevalence of 15-20%, of which the incidence of RPL comprised approximately 2-5% of the total pregnancy (2). The pathogenesis of RPL is a complex process comprising abnormal chromosome structure and function, immune dysfunction, endocrine disorder, abnormal reproductive structure, maternal factors, prethrombotic state, and the environment, among others (3, 4) (**Figure 1**). Although concentrated research has identified numerous causes for RPL, the etiology and pathogenesis of about 50% of RPL cases remain unclear. Such cases are referred to as, unexplained recurrent pregnancy loss (UPRL). Exploring the pathogenesis of URPL and providing early intervention are critical in ensuring a higher RPL live birth rate and a better pregnancy outcome.

The development of epigenetics provides a new perspective for the pathogenesis of RPL. Epigenetics refers that cells maintain or alter gene expression in stable or heritable manner without changing DNA sequence, including DNA or RNA methylation, histone or chromatin post-translational modification (PTM), and non-coding RNA (ncRNA) regulation et al. Epigenetics plays a vital role in the occurrence and development of RPL through regulating the expression of key genes determining cell differentiation, proliferation and apoptosis, which has been passed on to future generations by intergenerational and cross-generational inheritance and affected their health. These genes are expected to be the potential biomarkers for the diagnosis and new targets for the treatment of RPL. Numerous epigenetic studies have found that RPL is associated with defects in DNA methylation, methylation modification, and other related epigenetic modifications. Imprinting barriers, gene expression disorders, spermatozoa defect, and immune imbalance induced by abnormal DNA methylation may affect the process of embryo implantation, growth and development, and ultimately lead to the occurrence of RPL. To provide a new direction for the diagnosis and treatment of RPL, this review elaborates on the role of DNA methylation in the pathogenesis of RPL and its regulatory pathways.

## DNA Methylation and Related Enzymes

**Figure 2:**
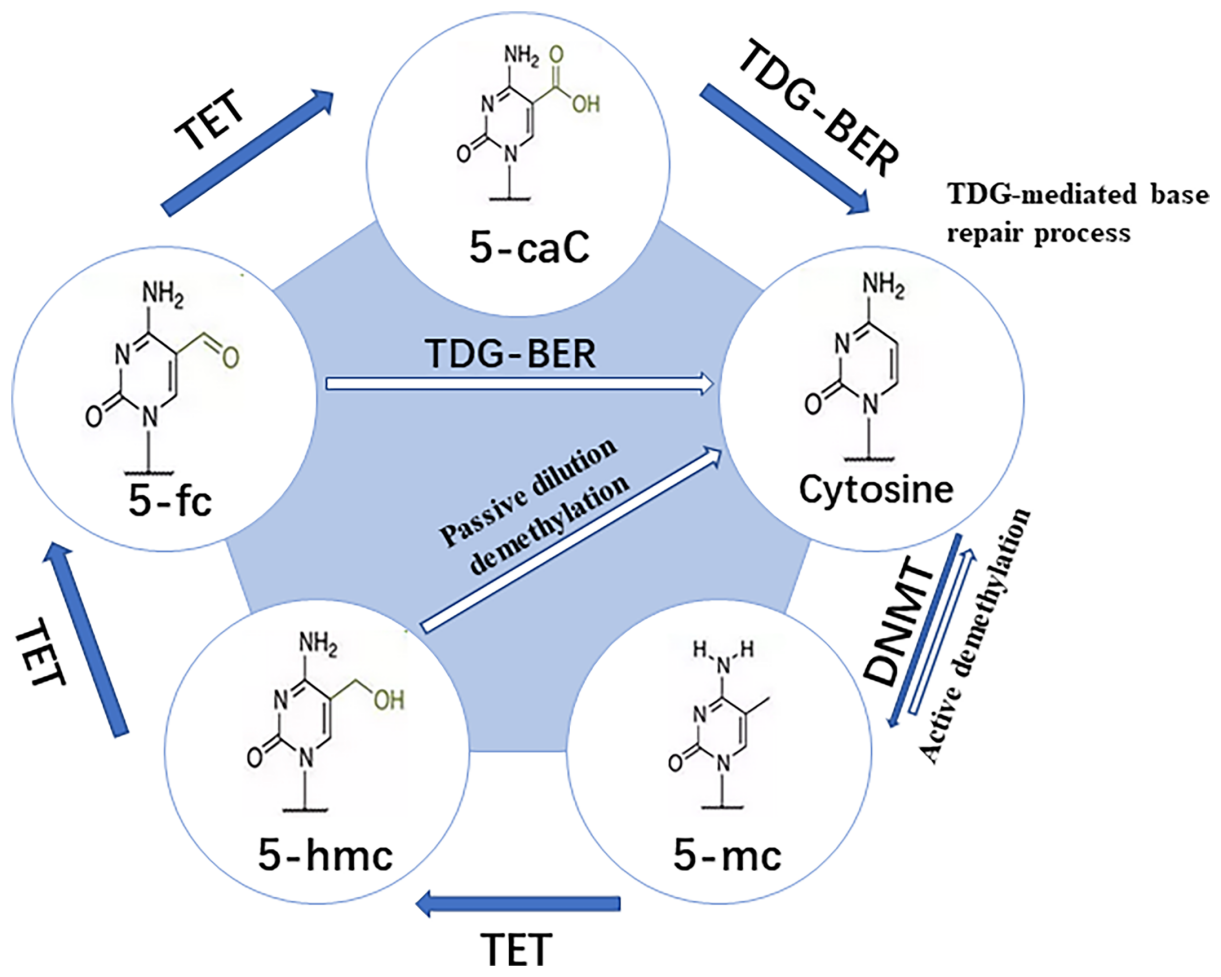
Pathway for dynamic modifications of cytosine. Methylation is achieved by DNMTs transferring the methyl to unmodified cytosine. It involves two steps: **(A)** Passive dilution, in which the methyl is directly removed to yield unmodified cytosine; and **(B)** Active demethylation, in which the methyl is catalyzed by TET into different oxidized forms and then combined with glycosylation, base repair, DNA replication, and other methods to yield unmodified cytosine (7).

DNA methylation is the earliest and most extensively studied epigenetic modification, which takes CpG Island as the central link. DNA methylation prevents the transcription factor (TF) from combining with DNA, or promotes the recruitment of methyl binding domain proteins for chromatin remodeling. It’s realized through generating 5-methylcytosine (m^5^C), which eventually leads to the silencing of gene expression (6). Extensive protein systems in cells write methylation patterns on DNA by *de novo* methylation (DNMT3a and DNMT3b) or demethylation (TET1, TET2 and TET3), and faithfully copy a series of factors (DNMT1 and UHRF1) of methylation patterns during DNA replication (**Figure 2**) (8). As an important epigenetic modification, the establishment, maintenance and clearance functions of DNA methylation play a key role in mammalian development and diseases (6). Previous studies have shown that DNA methylation is involved in the regulation of various biological processes. They include but are not limited to cell differentiation and development, gene transcription, chromatin structure remodeling, X chromosome inactivation, genome imprinting, and chromosome stability. At the same time, the pregnancy processes such as fertilization, embryo implantation, and placental development in human early embryo development are also closely related to DNA methylation (9, 10). The failure of cell invasion in early pregnancy, abnormal immune tolerance, and abnormal arterial remodeling of the decidua during placental development, which are controlled by a complex network of genetic-epigenetic modifications, may lead to the occurrence of URPL (9).

DNA methylation is mainly established and maintained by DNA methyltransferases (DNMTs), that transfer a methyl group from S-adenyl methionine (SAM) to the fifth carbon of a cytosine residue to form 5Mc. The enzymes include DNMT1, DNMT3a, DNMT3b and DNMT3L (11). DNMT1 is responsible for maintaining the DNA methylation pattern during the S phase in cell division (12). DNMT3a and DNMT3b initiate *de novo* methylation *in vivo* and are required for establishing new DNA methylation patterns during embryonic development (13). Although it lacks the conserved catalytic domain common to DNA methyltransferases, DNMT3L acts as a necessary accessory protein for DNMT3a-mediated *de novo* methylation in the germline (14–16). DNMT3c, as a tandem copy of DNMT3b, is only found in rodents, and it’s responsible for the DNA methylation of repetitive elements in the male germline (17, 18). All the DNMTs are extensively involved in the development of an embryo. It’s obvious that the deposition of methylation marks relies on the catalytic activity of DNMTs, while the active removal relies on the activity of ten-eleven translocation enzymes (TET) and thymine DNA glycosylase (TDG) (19). Various biological processes, including preimplantation of the embryo and PGC development, ESC maintenance and differentiation, neurological function, and cancer, have been shown to involve DNA demethylation (7). Research has revealed that global DNA demethylation of the preimplantation embryo genome is mainly accomplished through passive dilution and TET3-mediated oxidation (20, 21). In female mice, TET1 deficiency leads to PGC meiosis defects, which has been linked to insufficient demethylation and failure of meiotic gene activation (22). TET1 deficiency, on the other hand, causes abnormal methylation patterns at imprinted genes in PGC and sperm cells in male mice (23). In addition, TET1 and TET2 are both expressed in mouse embryonic stem cells (mESC) (24, 25). Deletion of TET1 manipulates ESCs towards specific lineages, while deletion of TET2 can inhibit enhancer activity and delay transcriptional changes during differentiation (26). TET1/TET2/TET3 triple knock-out (TKO) of ESCs has basically normal morphology and expresses pluripotency markers, but their differentiation and developmental potential are impaired (27). In summary, abnormal DNA methylation may change the embryo development potential, jamming embryo implantation and placenta formation, which may lead to adverse pregnancy outcomes.

## DNA Methylation Dynamics in Early Embryo Development

**Figure 3:**
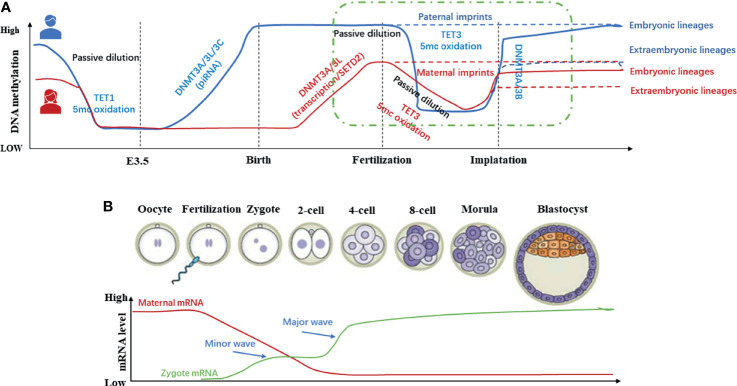
Methylation dynamics of gametes and early embryos during development (11, 29, 30). **(A)** In the germ line, DNA methylation in Primordial Germ Cells (PGCs) is almost completely eliminated to protect the PGCs from premature differentiation. Before mature gametes are formed, germ cells re-establish DNA methylation in a sex-specific manner. In early embryos, except imprinted genes and certain repetitive sequences, the genome undergoes global demethylation after fertilization and re-methylation after embryo implantation. **(B)** In general, global DNA methylation is mostly static throughout life. However, two waves of genome-wide DNA demethylation-remethylation occur in the germline and preimplantation embryos.

Methylation of DNA is a critical epigenetic regulatory mechanism in mammals during embryonic development. During gametogenesis and early embryo development stages in mammals, the whole genome undergoes a reprogramming event of demethylation-remethylation, realizing the continuation of the germline and the transmission of genetic material (28). Since 1987, it is believed that there are two demethylation-remethylation waves during development (**Figure 3**). The first waves of demethylation and remethylation occur during the migration of proliferating primordial germ cells and before the formation of mature gametes, respectively. During foetal life, the methylation marks on the imprinted genes are erased in the primordial germ cells. These marks are re-established during spermatogenesis up to the primary spermatocyte stage and faithfully maintained through the cell divisions by the DNA methyl-transferases. The second wave of demethylation occurs after fertilization, when the epigenetic memory of the gametes is erased on a large scale, and the lowest DNA methylation has been reached in the blastocyst (31). After embryo implantation, remethylation gradually occurs until it returns to the original high level (32). In other words, the methylation pattern of the early human embryo genome is reflected as follows: it’s at a high methylation level before fertilization, then the demethylation occurs during the cleavage stage, and the methylation level reaches a trough when the blastocyst is formed. Finally, it has been established a new methylation mode for interaction with the uterus after the embryo implantation to restore the original high methylation level. The periodic and orderly change of DNA methylation is the key to ensure the pluripotency of the developing embryo (33). Then, during the ovulation period, it is reflected through to the lowest level of methylation formed by the blastocyst, and finally the new methylation state in the mother-to-child interface. While researching on the reprogramming process of DNA methylation modification during preimplantation development of mouse cloned embryos, Gao Shaorong et al. revealed that abnormal DNA re-methylation is the key obstacle leading to abnormal development of cloned embryos after implantation (34). From this review and the existing literature, it can be suggested that the periodic and orderly changes in DNA methylation during early embryo development presents a novel research target of RPL.

## RPL and Abnormal or Defective DNA Methylation

DNA methylation runs through the entire reproductive process of gametogenesis, embryonic development, and maternal-fetal interface formation. Abnormal DNA methylation is a potential cause of early pregnancy loss (miscarriage or abortion within the sixth week of conception). Early embryo development (35), trophoblast cell invasion (36), maintenance of the uterine immune tolerance microenvironment, and uterine spiral artery remodeling are all controlled by a complex network of genetic-epigenetic modifications. Such modifications promote the occurrence of URPL. Globally, about 4% of spontaneous abortions have been linked to abnormal DNA methylation (37). Therefore, DNA methylation is an important molecular mechanism for the fertilization of the embryo and the normal development of the fetus. Vasconcelos et al. revealed for the first time, the disorder of epigenetic modifiers and imprinted genes in the placenta and fetal tissues of idiopathic spontaneous abortion. During the trimester, the author observed that TET2/3, IGF2, and CDKN1C were up-regulated; MEST was down-regulated; and methylation of MEST, KvDMR1, and H19 DMR was increased (38). DNA methylation may inhibit the expression of certain key genes in the embryo, which may interfere with the normal development and growth of the embryo, and promote the occurrence of RPL.

DNA methylation involves the selective addition of DNMTs and the selective deletion of TETs. Analysis of the chorionic villi and decidual tissues of URPL patients showed that they were hypomethylated and accompanied by down-regulation of DNA methyltransferases (DNMT1, DNMT3a, DNMT3b) and up-regulation of DNA demethylase (TET1/2/3) (39–42). The expression of DNMT1 and DNMT3a was significantly reduced in the receptive phase of the mouse endometrium. Clinical studies have revealed that the DNAMT3a (448A > G) gene polymorphism (43), and the DNMT3b gene polymorphism (rs1569686) may be a potential genetic marker of RPL susceptibility (44). Both DNMT1 and DNMT3a are found in the chorionic villus and decidua of normal early pregnancy. The interference of DNMT1 inhibitors may cause a decrease in the level of DNA methylation, impair embryo development, and inhibit the attachment between embryos and endometrial cells (45). Serman et al. found that after taking 5-Aza-CdR (DNA methylase inhibitor) in pregnant mice, the expression levels of DNMT1 and DNMT3a in the endometrial tissue decreased. Also, the proliferation of trophoblast cells was inhibited, and the embryo implantation rate was significantly decreased (46). Tanaka S et al. found that TET1 and TET2 regulate the expression of cyclin B1 through protein-protein interactions. As well, the enzyme regulate the integrity of trophoblast cells and the intracellular replication cycle, which affects the development of the fetus (47). In addition, demethylases (TET1/2/3) may induce the occurrence of URPL by interfering with the gamete meiosis, early embryo totipotency and differentiation of specific lineages (48, 49). We hope that through further experiments, we can find new means of preventing and treating URSA using methylation methyltransferase inhibitors.

Folic acid metabolism, as an important way to generate methyl donors (active methionine SAM), is inseparable from the occurrence of DNA methylation. the gene-specific methylation of 5,10-methykenetetrahydrofolate reductase (MTHFR) is a thrombophilic marker as well as it is positioned at the junction of the reactions producing methyl groups for the purpose of the DNA methylation, DNA repair and DNA synthesis (50). In RPL cases, MTHFR is significantly increased (p=0.002), which emphasizes the vital association between the methylation of MTHER and transformation (50). The clinical cases suggest that RPL’s spouse MTHFR (C677T/A1298C) polymorphism may alter sperm concentration and ratio of forward motility sperm (PR) by affecting DNA methylation, thereby increasing the risk of early spontaneous abortion (51, 52). In addition, the polymorphism of MTHFR (C677T) leads to low enzyme activity, changes the methylation cycle, and reduces the available free methyl. When these happen, they result in low global DNA methylation, which may be involved in the occurrence of preeclampsia (PE) and pregnancy loss (PL) (50). Rotondo et al. found that the promoter of MTHFR in the sperm of the RPL’s spouse showed a high degree of methylation. Based on the above researches, we suggested that abnormal methylation of MTHFR may affect the normal growth and development of the embryo, leading to the occurrence of RPL (53). We suspect whether simple supplementation of “folic acid” can compensate for the methylation defects in MTHFR-related RPL patients, thereby improving pregnancy outcomes? The feasibility of this measure requires further investigation of folate metabolism and other compensatory pathways in RPL patients.

## DNA Methylation of Imprinted Genes and RPL

Genomic imprinting is a process in which parental alleles undergo modification as they are passed on to offspring through gametes. Alleles with parental imprinting acquire different expression characteristics. During development, the methylation and expression status of imprinted genes are tissue and stage specific during development (54). More than 50 percent of known imprinted genes are expressed in placenta, and they are crucial to cellular differentiation and embryonic development (55). Abnormal DNA methylation in the imprint control area (ICR) of imprinted genes can lead to abnormal silencing of active alleles or abnormal expression of inactive alleles. This causes imprinting disorders or deletions, fetal neurodevelopmental defects and metabolic disorders, which in turn affect embryo development and induce poor pregnancy outcomes. Previous studies have reported that imprinted gene defects lead to early pregnancy loss, fetal death, prolonged delivery, and the development of embryo tumors (56–59). Liang et al. found that the maintenance level of the imprinted gene PEGIO is very important for early embryo development, whose down-regulation leads to irreversible pregnancy loss (60). Zhang et al. verified that the changes in the expression of imprinted genes and the hypermethylation profile of the repetitive regions (PRE-1 and satellite DNA) in cloned pigs may be related to the developmental defects and early pregnancy loss of the cloned pigs (61). Clinical studies have supported that PHLDA2, a maternal gene, is up-regulated in the placental tissues of the RPL, and inactivation of Mash2/Peg10 can cause abnormal placental development, which may lead to early embryo death. Flisikowski et al. reported that a microdeletion of PEG3 (a maternal imprinted gene of cattle) enhanced inhibition of the paternal gene MIMT1, late pregnancy loss, and even stillbirth (62). In addition, the expression of IGF2/PHLDA2/PEG10/CDKN1C was increased in first/mid-pregnancy abortion, while PEG10 was lowered in late pregnancy abortion or stillbirth (63). A series of clinical trials have also verified the hypothesis that “improper methylation and expression patterns of imprinted genes may lead to RPL” (64). While evaluating the DNA methylation patterns of the maternal genetic imprinting gene GRB10 and the paternal inherited genes (IGF2 and PEG3) in the villus samples of RPL, Liu et al. reported that both showed significant high methylation (64). PEG3 plays a key role in p53-mediated cell death and offspring development. Deletion and mutation of PEG3 may lead to insufficient placental nutrient transport and retardation of offspring growth, which may trigger offspring death (65, 66). IGF2 participates in glycogen production and affects placental metabolism or fetal growth. The quality of the fetus and placenta in placenta-specific IGF2-knockout mice is significantly reduced (67). The transgenic mice with the proximal duplication of chromosome 11 where GRB10 is located, exhibited pre-/post-natal growth retardation (58). To summarize, the defects of imprinting genes related to abnormal DNA methylation can promote the disorders of fetal neurological, developmental, metabolic and jamming embryo development. The placenta is an important organ for the enriched expression of imprinted genes. The methylation of the imprinted gene (H19/PEG1/LIT1/SNRPN) in the placental villi of RPL was significantly higher than normal (68, 69). The abnormal increase of H19/ICR1 methylation in the villi indicates that the methylation changes at special sites in the placental villi may be closely related to the risk of RPL (42). DNMT3L acts as an important structural protein that coordinates DNMT3A/DNMT3b-mediated DNA methylation during embryo development. The DNMT3L-knockout oocytes were unable to establish maternal-specific methylation imprints, resulting in abnormal fetal neural-tube development, which manifested as SA in the second trimester. It’s also been proven that DNA methylation is the key to genomic imprinted markers (70). Sheng et al. obtained E7.5/E13.5/E19.5 placentas from pregnant C57/BL mice, and screened the imprinted LncRNA controlled by DNA methylation during placental development. The results showed that the methylation of the MEG8 promoter increased in RPL villi, and the abnormal up-regulation of lncRNA MEG8 (RIAN) in the trophoblast cell inhibited cell proliferation and invasion. The author linked this observation to the occurrence of URPL (71). All in all, genetic imprinting disorders or deletions related to abnormal DNA methylation may promote the occurrence of RPL by interfering with embryo development (neural-tube development and metabolism), placental nutrient transport, trophoblast proliferation and invasion (72). We believe that in cases of RPL especially (unexplained RPL), early finding of new prognostic marker would be of great help to obstetricians for early detection and management of RPL patients.

## Sperm Abnormal DNA Methylation and RPL

RPL is also associated with certain male factors, including age, abnormal semen parameters, and sperm DNA damage. There is a growing body of evidence to support the hypothesis that the intact paternal epigenome is essential for normal embryo development (73). As the main epigenetic modification to maintain the normal development of sperm, DNA methylation can correctly compress the chromatin of the sperm head and permanently silence the gene promoters related to gene imprinting (22). Some studies have reported that the integrity and compactness of sperm chromatin in the RPL group are lacking (74–77). The abnormal DNA methylation of sperm interferes with spermatogenesis and embryonic development, leading to improper implantation and RPL (73, 78, 79). Sperm global methylation levels were found to be significantly reduced in the RPL group (80). The promoters of development-related genes in mature sperm often exhibit low methylation. The role of sperm DNA methylation in mammalian embryo develpment has been described by several authors. To identify the mechanism of how DNA methylation impacts sperm function and embryo development, several studies were performed in animal models. The earlier rodent studies have demonstrated that decreased global methylation levels in spermatozoa are associated with post-implantation embryo loss (81). DNA methyl-transferase family of proteins promote methylation and its maintenance (82). Studies in knockout mice and mutations in these proteins demonstrated an association with chromosomal deactivation, embryo growth interruption, and sub-sequent demise (12, 83, 84). In addition, studies that have used potent DNA methylation inhibitors in mice demonstrated an increased incidence of embryo mortality (85). It should be noted that embryo loss could be as a result of toxic effects of these inhibitors and not directly as a result of inhibition of DNA methylation (86). Furthermore, a recent study by Rogenhofer et al. demonstrated that P1 and P2 mRNA levels as well as the ratio of these two protamines were significantly different in male partners of women with unexplained recurrent pregnancy loss compared with healthy control men and subfertile couples undergoing *in vitro* fertilization/intracytoplasmic sperm injection (87). Based on the current body of evidence, we believe we can hypothesize that sperm epigenetics impact embryo development and alterations can be implicated in pregnancy loss. We await further studies on this idea.

Ankolkar et al. found that the methylation of imprinted gene H19 in the semen of the URPL spouse was significantly lower than normal, while there was no obvious difference in the methylation of PEG1/DLK1/GTL2/PLAGL1. Khambata et al. proposed several imprinted genes with abnormal DNA methylation in the sperm of URPL’s spouse that are related to RPL, such as IGF2-H19 DMR, intergenic differentially methylated region (IG-DMR), mesoderm specific transcript (MEST), zinc finger protein regulateing apoptosis and cell cycle arrest (ZAC, also called PLAGL1), DMR KCNQ1 intron 10 gene (KVDMR), paternally expressed gene 3 (PEG3) and paternally expressed gene 10 (PEG10), as well as decreased sperm global 5-methylcytosine (5mC) levels, are all related to RPL (80). IGF2-H19 DMR is an effective mitogen regulating the growth of embryos and placenta (88). The loss of DLK1 does not lead to embryo death, mainly manifested by defects in placenta formation and adipocyte differentiation (89). MEST, expressed in human villi and invasive trophoblasts during early pregnancy, contributes to angiogenesis during trophoblast invasion (90). PLAGL1 participates in the regulation of cell cycle, apoptosis and embryo development, whose lack may lead to embryo growth restriction and lethality (91). A meta-analysis showed that the hypomethylation and demethylation of H19 affect sperm concentration and motility (92, 93). The global 5mc level of sperm may be regarded as a diagnostic marker for RPL to detect “epigenetically abnormal” sperm, and the imprinted genes such as IGF-H19 will be the best candidate genes. In addition, MTHFR polymorphic variants are associated with decreased sperm count (53, 94, 95), leading to male oligospermia, infertility, and RA. Rotondo et al. believe that the hypermethylation of the MTHFR promoter in the sperm of URPL’s spouse may cause RA by abnormal embryo development and trophoblasts proliferation/apoptosis/differentiation (96). In conclusion, the paternal epigenome impacts sperm quality, early embryogenesis, and possible somatic health in future offspring (78). We need more transgenera-tional studies focused on environmental exposures in the F0 population, effects on sperm epigenetics, and any evidence of an impact on somatic health outcomes in offsprings. This will ultimately provide a path to preventative measures, diagnosis, and treatment.

## DNA Methylation of Placenta/Decidual and RPL

The placenta is the first organ formed during pregnancy, which plays an important role in the intrauterine regulation of fetal growth. The placenta plays multiple functions, such as the exchange of nutrients, waste, and gas, maternal-fetal immune tolerance, and various kinds of metabolic and endocrine functions during fetal development (97). Placental insufficiency may cause preeclampsia (PE) or intrauterine growth restriction (IUGR). The inflammation or infection in the placenta can lead to chorioamnionitis (CA) or placental-related preterm birth (PTBs). Tumor formation in the placenta is also called choriocarcinoma (CA) or hydatidiform mole (HM) (54). Epigenetic modification is believed to play a vital role in placental function and development (4, 98). In early pregnancy, the human placenta has similar phenotypic characteristics and epigenetic patterns to tumors, such as extensive hypomethylation of the whole genome and local hypermethylation of CpG islands (99). The epigenetic regulation of imprinted and non-imprinted genes is important in the placenta, date from the preimplantation stage and maintaining it throughout the whole pregnancy (54). Furthermore, DNA methylation may be inherently more variable in the placenta, because of its need to be responsive to a variety of signals in its function as a mediator of exchange between the fetus and mother (100). Thus, the placenta acts as the most important organ for the expression of imprinted genes. Lim et al. used RRBS (bisulfite sequencing) and RNA-seq to perform DNA methylation and gene expression analysis on human placental tissue. The author suggested that significantly altered genes were enriched in pathways related to the cell cycle and immune response (101). It is worth noting that human chorionic gonadotropin (HCG), whose coding genes CGB5 and CGB8 carry imprinting sites of placental expression, is an important hormone for embryo development and pregnancy maintenance. When the CGB5 promoter is hemi-methylated in RPL, the secretion of HCG is reduced, and the abortion rate is significantly increased (22). In a study that involved use of BSP to detect the methylation of the villi in RPL, Hannah et al. reported that RPL is closely related to the DNA methylation of specific sites in the placenta (like the decreased defensin β1 and the elevated AXL) (102). Elsewhere, Du et al. compared and analyzed the genome-wide DNA methylation of placental villi in RPL. The authors proposed that the hypomethylation of the PRDM1 promoter induces the recruitment of GATA2-FOXA1complex, promotes the migration and apoptosis of trophoblast cells, and leads to the occurrence of RPL (70). PRDM1 is a transcriptional regulator of embryo cell fate, required for reprogramming of primordial germ cells and intestinal cells (103). Wu et al. analyzed the LncRNA expression profile by microarray, and proposed that the abnormal methylation of IGF2AS in the placental villi may affect the stability of early pregnancy (104). Previous studies have shown that the formation and localization of the placenta may be the result of a balance between matrix metalloproteinases (MMPs) and tissue inhibitors of matrix metalloproteinases (TIMPs) secreted by the extracellular matrix. That means the expression of MMPs/TIMPs and the changes in the methylation of the promoter lead to the imbalance of MMPs/TIMPs and the occurrence of URPL (39, 104). A clinical study suggested that changes in placental DNA methylation of genes involved in environmental adaptation, immune response, and imprinted genes may contribute to the etiology of RM (45). However, it is difficult to determine whether these changes are causal or a consequence of placental adaptation to an unhealthy embryo. Furthermore, studying DNA methylation in the placenta is complicated by the presence of different cell types which carry their own unique methylomes. The subsequent research should reinforce the need to focus DNA methylation sequencing experiments on individual cell populations rather than entire tissue extracts.

Decidua, the maternal part of the placenta, provides a delicate balance between immune tolerance and defense to maintain pregnancy (105). The establishment and maintenance of normal pregnancy is accompanied with the proliferation, migration and invasion of trophoblast cells and endometrial decidualization. The invasion disorders or excessive invasion of trophoblast cells and abnormal endometrial decidualization can cause RPL. The endometrial decidualization involves the endometrial stromal cells’ reprogramming, the production of various mediators such as cytokines and chemokines, and the selective recruitment of immune cells. As an important part of the maternal-fetal interface, decidua plays a crucial role in the establishment and maintenance of pregnancy. First, it limits the excessive invasion of trophoblasts cells to protect the maternal uterus (106). Second, decidual secrete lipid droplets, glycogen, and growth factors provide nutrition for embryo development (107). Third, decidual enhances the construction of the maternal-fetal immune tolerance microenvironment to maintain normal pregnancy (108). Abnormal DNA methylation interferes with the expression of decidual related genes, leading to a decrease in immune tolerance, cell invasion failure, and poor remodeling of the uterine spiral artery. When these happen, improper implantation, inflammation, and impaired placental development may occur (109). DNA methylation analysis of the decidual in RPL showed that overexpression of CREB5 (cAMP responsive element binding protein 5) and SGK3 (SGK is a serine-threonine protein kinase regulated by glucocorticoids, involved in epithelial ion transport and cell survival) (110) can promote the occurrence of RPL by interfering with trophoblasts migration, apoptosis and dysfunction (111). The GO analysis suggested that hypo-DMR near CREB5could up-regulate CREB5 gene expression by recruiting P53 and SP1, thus increasing cell migration and cell apoptosis, blocking cell cycle (22). In addition, some studies have shown the function of CREB5 in the immune response, including macrophage survival, regulation of T/B lymphocytes, and inducing the transcription of immune-related genes (such as IL-10) (112). That means the hypomethylation of CREB5 may promote the occurrence of RPL by regulating the immune response. Chen H, et al. constructed a miRNA-mRNA network in decidual and identified several small molecules involved in the occurrence and progression of URPL, such as FCGR1A/3A (important receptors for innate and adaptive immune responses), CXCL8 (also known as IL-8, an important inflammatory factor), HCK [expressed in bone marrow cells, B lymphocytes, and various cancer cells to enhance the expression of myelin growth factor and pro-inflammatory cytokines (113)], PLEK [an important substrate of protein kinase C, the phosphorylation of PLEK can promote the secretion of pro-inflammatory cytokines in phagocytes (113, 114)], IL10 [low IL-10 are associated with pregnancy complications, such as RA, premature delivery, fetal premature membrane rupture, PE, IUGR (115)], hsa-miR498 and hsa-miR-4530 (116). A retrospective observational case-control study that revealed the succinate dehydrogenase complex iron-sulfur subunit (SDHB) DNA methylation, SDHB expression increased, and succinate level decreased in the decidua of RPL. Low accumulation of chorionic succinic acid interferes with the invasion and proliferation of extravillous trophoblast cells through the PHD2-VHL-HIF-1α pathway, and affects embryo implantation (117). Xie et al. found that the DNA methylation of lncHZ08 promoter was decreased in the decidua of RPL, and the estrogen receptor (ER)-mediating the transcription of lncHZ08 was increased. The up-regulated lncHZ08 inhibits the PI3K/p-AKT/p-P21/CDK2 pathway by down-regulating PI3K, thereby reducing the proliferation, migration and invasion of trophoblast cells, and further inducing the occurrence of abortion (118). Fatima et al. analyzed the causal relationship between methyltransferase, methylation and cell apoptosis in abortion through structural equation modeling (119). Studies is the first to show that the methylation of p53 pathway (BAX/P53/CASPASE-6/BCL-2) achieved by methyltransferase (G9aMT/DNMT1) is closely related to the fate (maintenance or termination) of early pregnancy (119). It is worth noting that the abnormal response of human endometrial stromal cells (HESCs) to decidual signals is closely related to RPL. The MEDIP-Seq analysis of HESCs suggests that RPL is significantly associated with the decrease in methylation of CA-rich sequences, which are widely expressed throughout the genome and enriched in near telomeres. This study suggested that abnormal DNA methylation of HESCs in the uterine can cause them to lose their epigenetic stemness, promote stromal cell senescence, limit endometrium plasticity, prevent decidua, and further induce RPL (120). HESCs *in vitro* experiments have shown that the loss of DNMT3b in DSC can down-regulate the expression of decidual-specific IGFBP-1. This implies that inhibiting DNA methylation can eliminate decidualization before or after implantation, leading to pregnancy loss (121). The DNMT1 and DNMT3a in the endometrium of the mice injected with DNMT inhibitors (5-Aza-CR) at different pregnancy stages were down-regulated, and the subsequent endometrial decidualization and stromal cell proliferation defects showed the potential to directly reduce embryo implantation rate (116).

## The Maternal-Fetal Immune Tolerance Microenvironment Is Regulated by DNA Methylation, As Is RPL

The endometrium is often regarded as an allograft that the embryo directly contacts. The main function of the endometrium is to create and maintain an optimal endocrine/paracrine, immune and molecular environment for proper attachment, implantation, invasion and development of the embryo. During pregnancy, the endometrial stromal cells undergo decidualization, a significant morphological and functional reprogramming of cells, cell reprogramming, tissue remodeling, gene expression, post-translational regulation, and change in the cell signal pathways. In the uterus, the activity of immune cells also undergoes major changes.

The maternal-fetal interface is an immune tolerance microenvironment composed of maternal decidua and fetal placenta, which are critical in ensuring the smooth progress of pregnancy. In this section, the composition of immune cells and related DNA methylation changes at the maternal-fetal interface of RPL is reviewed. Also, the effect of DNA methylation on the uterine immune microenvironment and the occurrence of RPL are explored.

The maternal-fetal immune interaction plays an important role in embryo implantation and pregnancy maintenance. Some scholars have proposed that URPL is a type of allogeneic immune disease related to the failure of maternal-fetal immune tolerance, which means the success of pregnancy depends on the immune status of the gravida and the immune regulation ability of the embryo (122). Du et al. performed a genome-wide DNA methylation analysis on the placental villi of RPL and found many significant DMRs near dysregulated genes (such as PRDM1) in RPL. These differentially expressed genes are enriched in the immune response pathway, which implies that abnormal immune regulation may promote the occurrence of RPL (70). The imbalances of the immune microenvironment at the maternal-fetal interface are usually reflected in two aspects (123). One is the lack of negative signal activation to maternal-fetal tolerance, which impairs embryo implantation or embryo formation. Excessive immune activation, on the other hand, increases the uterine inflammatory environment or causes trophoblast damage. Chuang G et al. carried out a study to identify the complete cell lineage of the decidual immune microenvironment of RPL through single-cell high-throughput sequencing. They documented several cells including but not limited to natural killer cells, macrophages, dendritic cells and T cells (124).

## Endometrial Stromal Cells Regulate Local Immune Function

Previous experiments have shown that proper decidualization of endometrial stromal cells plays an important role in implantation and maintenance of early pregnancy. The profound morphological and functional reprogramming of the endometrial stromal cells that differentiate into highly specialized cells with secretory capabilities, have been found in this progress. The overall emerging picture is that decidualization of the endometrium is a process involving profound cell reprogramming, tissue remodeling, changes in gene expression and post-translation regulation, and alterations in cell signaling pathways (125). These studies indicate that the endometrium is an excellent biosensor for the quality of implanted embryos (126–128). Normal female endometrial cells become sensitive to embryo signals after decidualization, thus low-quality embryos inhibit the secretion of factors beneficial to embryo implantation. On the contrary, embryos with strong developmental potential will produce signals promoting implantation (129). However, the RPL is not sensitive to this signal, allowing low-quality embryos to be implanted, leading to poor pregnancy outcomes (120).

## Macrophages

There are few studies on macrophages in the progression of RPL. The number of macrophages in the non-pregnant state is relatively small, only significantly increased in the luteal phase. Macrophages, rapidly increased in pregnancy, are located close to invading trophoblast cells and remodeled uterine spiral artery (120). They participate in embryo implantation, trophoblast invasion, remodeling of the uterine spiral artery, removing apoptotic cells and cell debris, and protecting the fetus from microorganisms or pathogens (130–132). A clinical study aimed at determining the DNA methylation group of macrophages on the maternal-fetal interface (133) reported as follows: First, there are significant differences in the DNA methylation patterns of macrophages derived from maternal or fetal cells. Second, differentially methylated genes related to immune response are highly methylated in fetal-derived macrophages. The imbalance between decidua M1/M2 macrophages is related to abortion (134). M1 macrophages increase the inflammatory advantage by producing inflammatory cytokines such as TNF-α, IL-β, IL-6 and IL-12, whereas M2 macrophages promote immune tolerance by producing anti-inflammatory cytokines such as IL-10 and TGF-β (135, 136). Contrary to normal pregnancy, we can’t detect the reduction of M1 decidual macrophages in URPL cannot be detected (134). Although the current research on DNA methylation of macrophages in the uterine microenvironment of RPL is still blank. However, a MeDIP-seq of preterm placenta demonstrated that abnormal DNA methylation was enriched in Fcy receptor-mediated macrophage phagocytosis (48), suggesting that abnormal DNA methylation in macrophages may be involved in adverse pregnancy outcomes.

## Natural Killer Cells (NK Cells): Are NK Cells the “BAD BOYS” That Reject Embryos?

As a member of the innate immune system, NK cells were originally defined as a cell population capable of killing autologous and allogeneic target cells through spontaneous cytotoxic activity. Based on the phenotype and function, NK cells can be divided into various subsets, such as peripheral NK (pNK) cells, uterine NK (uNK) cells, tissue-resident NK (trNK) cells and innate lymphoid cells-1 (ILC1s). This review focuses on uNK cells, mainly CD56^bright^ cells, with low cytotoxicity, but they can secrete rich cytokines to protect the alloantigen and regulate the process of pregnancy (137). The uNK cells reach a peak in early pregnancy and are adjacent to the invading trophoblast cells (138, 139). Studies have shown that activated uNK can produce angiogenic factors (VEGF and ANG2) and a large number of cytokines (GM-CSF, CSF-1, TNF-α, IFN-γ, TGF-β, LIF, IL2, CXCL10, CXL12) (140). During pregnancy, uNK cells play a unique role in regulating trophoblast cell invasion (141, 142) and remodeling the uterine spiral artery (123), which is the necessary step for placenta formation. In addition, the uNK plays an immune role at the maternal-fetal interface, and assumes the responsibilities of immune killing (such as killing microorganisms to avoid various inflammations) and immune tolerance (such as normal pregnancy) at the same time (143, 144). A series of clinical studies have demonstrated that compared with normal pregnant women, the number, quality and cytotoxicity of uNk cells of URPL are disparate (145, 146). The dysregulation of uNK cells in RPL may cause pregnancy damage through the following five potential ways: (1) NK cell cytotoxicity is maintained but impaired (147); (2) the ability of NK cells to correctly interact with the specific HLA expressed by trophoblasts is impaired (148, 149); (3) the ability of NK cells to effectively participate in the complete uterine spiral artery remodeling is impaired (150, 151); (4) the limitation of T cell cytotoxicity is impaired and (5) NK cells interferes with the mode of cytokine production (152–154). To date, the fact that NK cells are related to RPL is well known, but its related epigenetic regulation mechanism is still unclear. Whether the proportion, functional status and methylation level of NK cells are related to RPL remains to be further explored.

## The Balance of Th1/Th2 or Th17/Treg and RPL

In 2010, Saito et al. proposed that the Th1/Th2/Th17 and Treg cell paradigms play a crucial role in maternal immune tolerance (155). Th1 cells contribute to cellular immunity by producing IL2 and INF- α, which are thought to be the basis for allograft rejection (156, 157). On the contrary, Th2 cells release IL4, IL5 and IL13 and participate in humoral immunity (158), as the key to inducing and maintaining allograft tolerance (159). Th1 cells secrete cytokines (IL2/TNF-/IFN-), participate in immune surveillance, and protect against excessive trophoblast invasion (160). A pro-inflammatory Th1 immune response is required to promote tissue remodeling and angiogenesis during embryo implantation (149). Comparatively, pregnant women having RPL have higher Th1 levels in their peripheral blood than normal pregnant women (161). Th1 and NK cells secrete a large amount of TNF-α, which causes apoptosis, inhibits trophoblast growth, and inhibits the uterine epithelium’s secretion of granulocyte macrophage colony stimulating factor, ultimately leading to pregnancy loss (162, 163). Once the implantation period is over, the immunodominance of Th1 cells gradually shifts to Th2, participating in maintaining the maternal tolerance to fetal antigens (164) until delivery. Th2 immunity represses the development of Th1 and Th17 immunities by releasing IL-4 and IL-13, respectively, and promotes allograft tolerance (165). Meanwhile, Th2 cytokine, IL-4, was reported to induce autoreactive B cell activation and thus promotes autoimmunity (166). The exacerbated Th2 immunity during pregnancy may induce autoimmune diseases (systemic lupus erythematosus) (167), while the tolerogenic may induce uncontrolled viral infections (ZIKA virus) (168). Timely and adequate Th2 immunity is important for the immunotolerance and protection of fetus from infection. Therefore, the timely transfer and adequate balance of Th1 and Th2 cells during pregnancy seem to be essential for a successful pregnancy. Conversely, an improper balance during pregnancy is related to pregnancy complications, such as RPL and PE. Reversing the Th1/Th2 imbalance in RPL patients and supplementing the lack of related cytokines can provide new ideas for the targeted therapy of RPL. In the LPS-induced abortion mouse model, Administration of IL-10, which has immunoregulatory properties, and TNF-α receptor blocker etanercept, prevented LPS-induced pregnancy losses (169). When shifted Th1 immunity was regulated with intravenous immunoglobulin G (IVIg), TNF blockers, or T cell activation inhibitors, such as etanercept, adalimumab, or tacrolimus, pregnancy outcome was significantly improved in women with RPL (170–174).

Th17 cells, producing effective pro-inflammatory IL17, play an important role in inducing inflammation and immune rejection (175). The interaction between Th17 and Th1 is related to the pathogenicity of allergies, autoimmune diseases, immune rejection and adverse pregnancy outcomes (49, 176–179). In addition, Th17 can induce the activation of dNK cells and impair the vascular reactivity of the uterine artery (160), leading to embryo resorption (49, 180). The excessive Th17 cells were detected in the decidua and peripheral blood of unavoidable abortion (177, 181–183). Treg cells play an important role in embryo implantation and early pregnancy as participants in mediating maternal-fetal tolerance (162, 163). The proliferation of Tregs is related to normal pregnancy, while the reduction promotes embryo immune rejection and induce pregnancy loss (163). In animal models, the consumption of Tregs significantly increases the abortion rate (184, 185). Conversely, the transfer of Tregs can prevent pregnancy loss in miscarriage-prone mice (186). Similarly, in clinical trials, compared with normal pregnant women, the Tregs of the peripheral blood (187, 188), endometrium and decidua (189, 190) in RPL have been reduced, accompanied by increased Th17 cells (190, 191). The possibility of Tregs involved in RPL is supported by the key transcription factor FOXP3. Wang et al. found that the hypermethylation of Foxp3 promoter down-regulates the expression of the FOXP3, reduces the number of Tregs, leads to the imbalance of Treg/Th17 in the maternal-fetal immune interface, and promotes the occurrence of RPL (192–195). At the same time, Treg cells can participate in the down-regulation of maternal-fetal excessive inflammatory response and embryo implantation, whose imbalance or dysfunction may lead to the occurrence of RPL (189). Finally, the highly malleable Tregs can differentiate into effector T cells under certain conditions. Unbalanced Tregs may directly participate in fetal rejection (196). Although there is still a lack of direct evidence to confirm the driving effect of abnormal DNA methylation of Th cells and Treg cells in RPL. However, the existing research suggests that abnormal DNA methylation may promote the occurrence of RPL through regulating the proliferation, differentiation and cell activity of Th cells and Treg cells. Detecting the proportion of Th cell population at the maternal-fetal interface and intervening in Th cell activation is beneficial to the prevention and treatment of RPL patients.

## Imbalanced Cytokine-Chemokine Network in RPL

Cytokines, chemokines and their receptors can be produced by endometrial glandular epithelial cells and stromal cells, decidual cells, cellular components of innate and adaptive immunity, and trophoblast cells. This network creates a local immune balance microenvironment, which is essential for successful embryo implantation, the regulation of trophoblast migration, and a normal pregnancy. In RPL patients or mouse models, the dysregulation of cytokines associated with embryo implantation and early pregnancy is present in or recruited to immune or non-immune cells in the endometrium and decidua. In turn, the unfavorable cytokine environment will impair the tolerance of the maternal immune system to the trophoblast, leading to immune rejection. RPL is related to the decrease of TGF-β produced by decidual dendritic cells (197). The increased IFN-γ in the decidua of RPL can induce apoptosis and embryotoxic effects and promote the occurrence of RPL by triggering the excessive inflammatory response of the decidua (198, 199), IL-10, like TGF- and IDO, can induce DC differentiation into a tolerance phenotype and regulate Treg population expansion. The reduction of IL-10 in the decidua of RPL will hinder this immune protection (200–202). In view of the complexity, sensitivity and plasticity of the endometrial cytokine-chemokine network, it is still difficult to determine all the cytokines involved and their cell sources in RPL. The mechanism and specific signaling pathways of the abnormal DNA methylation-induced cytokine-chemokine network imbalance in the maternal-fetal interface promoting the RPL are still to be further explored. The in-depth research of epigenetic regulation in immune regulation and related signaling pathways of RPL is expected to provide opportunities for personalized diagnosis and treatment of RPL.

In the last three decades, enormous progress has been made in the comprehension of the mechanisms establishing and maintaining the maternal-fetal interface immune tolerance microenvironment. The involvement of the maternal immune system is not limited to a correct immunologic dialogue at the maternal–fetal interface, but is extended to the endometrial decidualization, vascular remodeling, and placentation. It’s obvious that the maternal-fetal immune tolerance microenvironment is intimately involved in the establishment, maintenance, development, and termination of the normal pregnancy. Pregnancy loss due to maternal immune dysfunction can be prevented by inducing an overall immunological tolerance at maternal-fetal surface and maintaining full immunological reactivity against all the other foreign antigens. In fact, the currently available immune treatments for RPL are rather limited, empiric in the majority of cases, and with low efficacy. On the basis of the existing researches, the future perspectives in the immune treatment of RPL could be aimed to correct abnormal decidualization as well as dysfunctions of the maternal-fetal immune tolerance microenvironment. Although there is still a lack of direct evidence and related mechanisms of DNA methylation regulating the maternal-fetal immune microenvironment. Considering the similarity between pregnancy and tumors, we expect that new anti-tumor therapy “epi-drugs” can also shine in RPL immunotherapy.

## Conclusion

Recurrent pregnancy loss (RPL) is one of the most frustrating and difficult areas in maternal-fetal medicine. The RPL rates have continued to rise during the last few decades, yet the underlying mechanisms remain poorly understood. An emerging area of interest is the mediation of essential gene expression by epigenetic modification during early pregnancy. Epigenetic intergenerational or cross-generational inheritance support “Fetal Origins of Adult disease (FOAD)”. Looking at it from this point of view, it may be considered lucky as the abnormal epigenetic load from the maternal side gets lost and not transferred to the next generation. The number of studies about DNA methylation has increased linearly since its discovery in the 1980s, while much remains unknown. For example, why do abnormal DNA methylation embryos die at such an early stage of development? How does abnormal DNA methylation lead to RPL? We are now in an era of unprecedented genetic tools, sensitive and highly quantitative sequencing technologies, and the ability to alter DNA methylation. All problems seem to be solved, but challenges still exist.

The pathogenic factors of RPL are complex and diverse. DNA methylation plays a key role in early embryonic development, trophoblast cell invasion, uterine spiral artery remodeling and maintenance of the maternal-fetal interface by precisely regulating the inheritance and expression of genes. This review summarizes the relationship between abnormal or defective DNA methylation and RPL, and describes four aspects (imprinted genes, placental/decidual genes, sperm DNA, and immune-related genes) in detail. This review focuses on the composition of immune cells and related DNA methylation changes at the maternal-fetal interface of RPL. Earlier, the effect of DNA methylation on the uterine immune microenvironment and the occurrence of RPL were explored. This review fills a gap in the study of the important role of epigenetic regulatory networks in embryo development and the maternal-fetal interface. In this part, more basic experiments are needed to conduct in-depth research on the molecular mechanism and related signal pathways of epigenetic regulation in RPL. We also need to fully explore the complex association network between different regulation methods and their mechanism acting in early embryo development, embryo implantation, and the maternal-fetal interface. Our review opens up new avenues to screen women prior to pregnancy for the risk of miscarriage and point to the potential of immune therapies based on epigenetics in the prevention of RPL.

## Author Contributions

QZ wrote the manuscript, made the figures, and critically reviewed the manuscript. YX and BQ wrote the manuscript and critically reviewed the manuscript. AB and YZ critically reviewed the manuscript. All authors contributed to the article and approved the submitted version.

## Funding

This work was supported by the following grants: National Key Research and Development Program of China (No. 2018YFC1004601), and the National Natural Science Foundation of China (No. 81801540, 81771662).

## Conflict of Interest

The authors declare that the research was conducted in the absence of any commercial or financial relationships that could be construed as a potential conflict of interest.

## Publisher’s Note

All claims expressed in this article are solely those of the authors and do not necessarily represent those of their affiliated organizations, or those of the publisher, the editors and the reviewers. Any product that may be evaluated in this article, or claim that may be made by its manufacturer, is not guaranteed or endorsed by the publisher.
